# Body image dissatisfaction is associated with perceived body weight among secondary school adolescents in Harar Town, eastern Ethiopia

**DOI:** 10.3389/fpsyt.2024.1397155

**Published:** 2024-05-17

**Authors:** Abainash Tekola, Lemma Demissie Regassa, Miheret Mandefro, Samrawit Shawel, Obsan Kassa, Feyisa Shasho, Teshome Demis, Awoke Masrie, Aklilu Tamire, Kedir Teji Roba

**Affiliations:** College of Health and Medical Science, Haramaya University, Harar, Ethiopia

**Keywords:** body dissatisfaction, body shape dissatisfaction, body appearance dissatisfaction, adolescent, body image

## Abstract

**Purpose:**

Body image dissatisfaction has emerged as an important determinant of dietary and physical activity, which in turn determine adolescents’ nutritional status. Hence, it is important to understand predictors of body image dissatisfaction. Therefore, this study aimed to assess body image dissatisfaction and its associated factors among secondary school adolescents in the study area.

**Methods:**

Data collected for other purposes were used to assess body image dissatisfaction among adolescents in Harar town. Body image dissatisfaction was assessed using the Body Part Satisfaction Scale. Data processing and analysis were performed using STATA version 14, and binary logistic regression was used to identify factors associated with body image dissatisfaction. Factors associated with body image dissatisfaction were determined by estimating AOR along with 95% CI and the statistical significance was declared at a p-value less than or equal to 0.05.

**Result:**

The prevalence of body image dissatisfaction was 22.06% [95% CI (19.63, 24.70)]. This study also revealed that males were more dissatisfied with their upper torso and face, and females were more dissatisfied with their middle torso, lower torso, height, and weight than males. In this study, body image dissatisfaction was associated with perception of being fat [AOR = 1.89, 95% CI (1.23, 2.91)], anxiety [AOR = 1.59, 95% CI (1.02, 2.48)], and cigarette smoking [AOR = 1.63, 95% CI (1.03, 2.58)].

**Conclusion:**

Almost one in five secondary school adolescents in Harar had body image dissatisfaction, which was significantly associated with perceptions of being fat, anxiety, and smoking. In this study, a significant number of the adolescents experienced body image dissatisfaction. Hence, all concerned bodies have to take action to reduce the burden.

## Introduction

Body image is a person’s perception of their physical self and the thoughts and feelings of positive, negative, or both, which result from that perception (1). Body image dissatisfaction (BID) can be defined as the negative attitudes, perceptions, beliefs, or feelings that one carries with respect to his or her own body (2). Comparing one’s own physical appearance to that of people on social media, as well as the drive for thinness among teenagers, leads to body dissatisfaction (3, 4). In addition, exposure to teasing because of body shape, as well as appearance criticism from friends and family, can generate internalized appearance ideals that predict body satisfaction levels (5–7). Thin-ideal internalization has a significant effect on body dissatisfaction for both genders. Thin-ideal internalization has a significant effect on body dissatisfaction for both genders. The desire to be thin was higher in girls, and boys aspire to have a larger body size; muscular-ideal internalization was associated with greater body dissatisfaction in boys (7–9).

BID arises throughout early adolescence due to rapid physical growth, psychological development, body self-consciousness, and societal expectations (10). Currently there is an increase in BID owing to the high use of social media and internalization of the aesthetic thin ideal (IATI), which underpins body dissatisfaction among both boys and girls (4, 5, 11). Previous studies show different distributions of BID among adolescents at different site. In a study conducted among adolescents in Brazil, 19.5% had body image dissatisfaction (12), 60.1% in Malaysia (13), 25% among girls, and 10% among boys in Indonesia (14), 75.2% in Kathmandu in Nepal (15), 21.2% in Jordan (16) and 21.6% in Egypt (17).

Although adolescents with normal body weight may also be dissatisfied with their bodies, it is higher among overweight/obese adolescents, and dissatisfaction is an internal process influenced by several factors. External factors such as social media exposure and negative appraisal can cause BID among adolescents (16, 18–20). Furthermore, it is associated with family conditions such as having unemployed fathers and mothers, a low level of maternal education, and a lower socioeconomic status (17). It is also associated with individual factors, such as body weight concerns and a desire to be thinner or larger (6). Even though adolescents with normal body weight may also be dissatisfied with their bodies, it is higher among overweight/obese adolescent and those who perceive themselves as fat (9, 10, 12, 13, 15, 17, 20). Furthermore, it is higher among those who engage in weight-control behaviors, those who exercise, follow a diet to lose weight, and those who have negative attitudes toward eating (6, 12, 17, 20). Body image dissatisfaction also associated with psychological problems. It is common among adolescents with depression and anxiety, and higher self-esteem is significantly associated with lower body dissatisfaction (4, 17, 20, 21).

Body image has emerged as an important determinant of dietary and physical activities (14). BID could change the meal patterns of the adolescents. Those who wished to be thinner had less than or equal to three eating occasions per day, skipped breakfast more frequently, and restricted food groups and energy intake, which affected nutritional status (22, 23). Dissatisfaction with one’s body has an effect on adolescent mental health, with a positive influence on the likelihood of reporting frequent feelings of loneliness, insomnia due to anxiety, and difficulty socializing (24) It also reduces self-esteem, which can have a negative impact on mental abilities and lead to the development of unhealthy coping skills such as unhealthy weight loss strategies and eating disorders (25, 26). In addition, body dissatisfaction predicted the risk of suicidal ideation (27). Generally body image satisfaction level is associated with the quality of life of an individual in many ways in both genders (28).

Even with evidence from developed countries, there is an information gap regarding the level of body image dissatisfaction and related factors in developing countries. To our knowledge, no study has examined the level of body image dissatisfaction among adolescents in Ethiopia, specifically Harar. Hence, this study primarily aimed to investigate the prevalence of, body image dissatisfaction among secondary school adolescents in Harar Town and the second objective of this study is to identify factors associated with, body image dissatisfaction among secondary school adolescents in Harar Town.

## Materials and methods

### Source of data and setting

This cross-sectional study used secondary data. The data for this analysis were obtained from previously collected data for the assessment of disordered eating among secondary school adolescents in Harar Town from June 30 to July 15, 2022. Harar town located 525 kilometers east of Addis Ababa (the capital of Ethiopia). The town contains 15 secondary schools, seven of which were public and the rest were private. For this study, eight schools (four public and four private) were chosen by lottery method. After that sections that had to be included were drawn at random from all sections in randomly selected secondary schools. Finally, all adolescents in randomly selected sections were included in the study. The study includes 1061 secondary school adolescents. Data were collected using structured, pre-tested, and self-administered questionnaires which was translated into Amharic and Afan Oromo.

### Sample size and sampling procedure

The sample size for this study was calculated using single population proportion formula. With expected prevalence of 21.6% from study conducted in Egypt (17) at 95% confidence interval and 5%, margin of error which yield minimum sample size of 260. Since the study used secondary data we have decided to use the overall data except those observations with missing data for the outcome variables. Finally, the current study included data of 1029 adolescents.

### Variables

#### Dependent variables

The outcome variable was body image dissatisfaction, which was measured using the body part satisfaction scale (BPS). Body part satisfaction scales assessed dissatisfaction with the face, upper torso, middle torso, lower torso, height, body weight, muscle tone, and overall appearance. The scale is graded on a six-point scale. One represents extreme satisfaction, two represents quite satisfaction, three represents some-what satisfaction, four represents some-what dissatisfaction, five represents quite dissatisfaction, and six represents extreme dissatisfaction (29). This scale is not validated in Ethiopia but it has been used in study conducted among adolescents in Ethiopia (30). In this study the Scale has highest internal consistency (α =.92).

#### Independent variables

The independent variables were socio-demographic factors, such as age, school type, sex, residence, father’s occupation, mother’s occupation, maternal educational status, and paternal educational status.

Aside from this, some other factors like body weight, meal skipping, level of exercise, perceptional body weight, anxiety, depression, stress, cigarette smoking, and exposure to media were also included as independent variables. Most of independent variables were assessed by self-report questions.

##### Depression, anxiety, and stress

Was measured using the Depression, Anxiety, and Stress Scale-21 (DASS-21). It is a set of three self-reported scales designed to measure the emotional states. The scale contains 21 items. Each of the three DASS-21 scales contains seven items (31). This scale is not validated in Ethiopia, even though it has been used in study conducted among adolescent in Ethiopia (32). It was classified as abnormal if the score was above 9, above 7, and above 14 for depression, anxiety, and stress, respectively (Antony et al., 1998). In this study, the scale has the highest internal consistency (**α** =.91).

##### Perceptional body weight

Was assessed by using the question, “How do you perceive about your body weight?” from four alternative responses: underweight, normal, overweight, and obese (Tuffa et al., 2020) (30).

#### Operational definition

##### Body image dissatisfaction

Individuals who rated their overall appearance satisfaction level as somewhat dissatisfied, quite dissatisfied, or extremely dissatisfied were considered as having body image dissatisfaction (29).

##### Depression, anxiety and stress

Individuals who scored above 9, 7, and 14 for depression, anxiety, and stress on the DASS-21 scale were classified as having depression, anxiety, and stress, respectively (31).

##### Body weight

The World Health Organization growth reference from 2007 was used to classify adolescents. Body mass index(BMI) for age Z-score <−2 as thin, ≥−2 and ≤+1 as normal weight, >+1 and ≤+2 as overweight/obese (33).

### Methods of data processing and analysis

To maintain data quality, 32 cases missing for the outcome variable were excluded. After computing the average weight and height for each individual, the anthropometric data were exported to the WHO Anthro Plus software to generate BMI for age. Data processing and analysis were performed using STATA version 14. Frequencies, percentages, medians, and interquartile ranges were used to describe variables. A binary logistic regression model was used to identify factors associated with body image dissatisfaction. The variance inflation factor (VIF) was used to test the independent variables for multicollinearity; no significant multicollinearity was observed (VIF < 10). The model fitness was checked using the Hosmer–Lemeshow test, and it fitted well with a p-value of 0.332. Factors associated with body image dissatisfaction were determined by estimating the AOR along with the 95% CI, and statistical significance was declared at a p-value ≤ 0.05.

## Results

### Socio-demographic characteristics

Data from 1029 adolescents were used in this study. Of the total participants, 554 (53.84%) were female, and the median age of the participants was 17 years with an interquartile range of 2 years. Of the total number of adolescents, 704 (68.42%) were from public schools and 845 (82.12%) lived in urban areas. Most adolescents were from families that attended at least primary schools. Regarding family occupation, most adolescents’ mothers 344(33.43%) were housewives and most of their fathers (290, (28.28%) were government employees (**Table 1**).

**Table 1:** Socio-demographic characteristics of Harar town secondary school adolescents and their families, July 2022.

Variables	Categories	Frequency	Percentage
Sex	Male	475	46.16
	Female	554	53.84
School type	Public	704	68.42
	Private	325	31.58
Residence	Urban	845	82.12
	Rural	184	17.88
Maternal educational status	No formal education	272	26.43
	Primary school	204	19.83
	Secondary school	283	27.50
	Technical/vocational	49	4.76
	Higher	221	21.48
Paternal educational status	No formal education	147	14.29
	Primary school	209	20.31
	Secondary school	295	28.67
	Technical/vocational	60	5.83
	Higher	318	30.90

### Body weight

A total of 834(81.05%) adolescents had a normal Body weight, and the rest were either underweight or overweight. Of these, 126(12.24%) were thin and 69(6.71%)were either overweight or obese. In this study, 56 (11.79%) of males and 70 (12.64%) of females were underweight, whereas 33 (6.95%) boys and 36 (6.50%) of females were either overweight or obese.

### Prevalence of depression, anxiety and stress

In this study 439(42.66%) of adolescents had stress, 633 (61.52%) had anxiety and 702(68.22%) had depression.

### Prevalence of body image dissatisfaction.

The prevalence of body image dissatisfaction was 22.06% [95% CI (19.63, 24.70)]. The prevalence was higher among females compared to males. This study also revealed that males were more dissatisfied with their upper torso and face than females, and females were more dissatisfied with their middle torso, lower torso, muscle, height, and weight than males (**Table 2**).

**Table 2:** Dissatisfaction with Each Aspect of the Body on the Body Parts Satisfaction Scale July 2022.

Body part	Male	Female
Face(facial features, complexion, hair)	86(18.11)	80(14.44)
Upper torso(chest or breasts, shoulders, arms)	128(26.95)	127(21.12)
Mid torso (waist, stomach)	121(25.47)	161 (28.15)
Lower torso (buttocks, hips, legs, ankles)	114(24.00)	146(26.35)
Muscle	95(20.00)	125(22.56)
Height	98(20.63)	143(25.81)
Weight	111(23.37)	165(29.78)
Over all appearance	98(20.63)	129(23.29)

### Factors associated with body image dissatisfaction

Bivariate and multivariate analyses were performed to identify factors associated with body image dissatisfaction. All variables with a p value ≤ 0.25 during bivariate analysis, were included in the multivariate analysis. This study showed that body image dissatisfaction was significantly associated with perceptions of being fat, anxiety, and cigarette smoking. The odds of body image dissatisfaction were 89% [AOR = 1.89, 95% CI (1.23, 2.91)] higher among adolescents who perceived themselves as fat than among those who perceived themselves as having a normal body weight. In addition, the odds of body image dissatisfaction were 63% higher among adolescents who smoked cigarettes [AOR = 1.63, 95% CI (1.03, 2.58)] compared among others. Additionally, the odds of body image dissatisfaction were 59% higher among adolescents with anxiety [AOR = 1.59, 95% CI (1.02, 2.48)] than among others (**Table 3**).

**Table 3:** Factors associated with body image dissatisfaction among secondary school adolescents in Harar town, eastern Ethiopia, July 2022.

Variables	Body image dissatisfaction		COR(95% CI)	AOR (95% CI)	P value
	Yes	NO			
Sex					
Male	98(20.63)	377(79.37)	1	1	
Female	129(23.29)	425(76.71)	1.17(0.87,1.57)	1.14(0.84, 1.57)	0.400
Perceptional body weight					
Normal	103(18.90)	442 (81.10)	1	1	
Underweight	77(22.32)	268(77.68)	1.23(0.88,1.72)	1.08(0.76 1.52)	0.672
Overweight/obese	47(33.81)	92(66.19)	2.19(1.45,3.31)	1.89(1.23 2.91)	0.004
Smoking cigarette					
No	190(20.59)	733(79.41)	1	1	
Yes	37(34.91)	69 (65.09)	2.07(1.34,3.18)	1.63(1.03 2.58)	0.038
Anxiety					
No	54(13.64)	342(86.36)	1	1	
Yes	173(27.33)	460(72.67)	2.38(1.70,3.33)	1.59(1.02 2.48)	0.042
BMI					
Normal	184(22.06)	650(77.94)	1	1	
Under	29(23.02)	97(76.98)	1.06(0.67,1.65)	1.15(0.72,1.84)	0.550
Overweight	14(20.29)	55(79.71)	0.90(.50,1.65)	0.86(0.46 1.62)	0.649

## Discussion

This study revealed that 22.06% [95% CI (19.63, 24.70)] of adolescents attending secondary school in Harar Town had body image dissatisfaction. In this study, body image dissatisfaction was significantly associated with the perception of being fat, anxiety, and cigarette smoking. Body image dissatisfaction is a common health issue among adolescents (10). Since adolescence represents a critical stage in the development of either a positive or negative body image, as a result of influences that exist during adolescence, including the transition to puberty, that affect one**’**s body shape, weight status, and appearance (34). There is a high prevalence of body image dissatisfaction among adolescents in developed countries, and there is little information about this problem in African countries. In Ethiopia, there is scarcity of research regarding this problem, and this study found a high prevalence of body image dissatisfaction among adolescents at the study site. This further shows that body image concerns are no longer problems for only developed countries; even in developing countries, the level of body image dissatisfaction is increasing rapidly among adolescents (35) So the Harar Town regional health office must emphasize this health issue by considering its consequences for adolescents.

The prevalence of body image dissatisfaction in this study is almost comparable to that of a study conducted in Jordan (21.2%) (16) Turkey (20.2%) (36) and Egypt (21.6%) (17) but it was lower than that in a previous study conducted in Malaysia (13) United Arab emirates (2) and Nepal (15). This difference in prevalence may be attributed to cultural differences, as most Ethiopians favor being fat in contrast to developed countries. This could also be due to differences in the assessment tools. A study conducted in Malaysia and Nepal used the Body Shape Questionnaire to assess body image dissatisfaction, unlike this study, which used the BPS. In addition, there can also be disparity within the population, as body image dissatisfaction is determined by different socioeconomic problems, which may result in differences in prevalence (17).

This study showed a slightly higher prevalence of body image dissatisfaction compared to a previous study conducted among adolescents aged between 11 and 17 years who were students in public schools in Brazil (12). This difference in prevalence may be due to differences in assessment tools.

In this study, the odds of body image dissatisfaction were higher among adolescents who perceived themselves to be overweight. This finding coincides with that of a previous study conducted in Brazil (12) and the United States (37), which showed that the prevalence was higher among adolescents who perceived themselves as fat. This could be due to the currently accepted idea that being thin is a sign of beauty, especially among adolescents. This significantly affects body satisfaction levels in both sexes (8). Therefore, health professionals must emphasize adolescents who perceive themselves as overweight to reduce the problem.

In this study, the odds of body image dissatisfaction were higher among adolescents with anxiety than among those without. The association between anxiety and body image dissatisfaction has also been shown in other studies (17, 38). This could be due to dissatisfaction with one’s own body, which may lead to low self-esteem and cause difficulty in socializing, which in turn has an effect on psychological distress (24, 38). This study also revealed that the odds of body image dissatisfaction were higher among adolescents who smoke cigarettes. This is in line with the study conducted in the UK that shows that body image dissatisfaction was associated with increased odds of smoking (39). This can be due to the fact that adolescents who have body image dissatisfaction may smoke cigarettes to escape the feeling they have about their body appearance. Therefore, to reduce the burden of this important health issue among adolescents, health professionals have to screen adolescents for body image dissatisfaction with smoking cigarettes, and vice versa.

Even though BMI did not significantly associate with body image dissatisfaction in this study, BMI was significantly associated with body image dissatisfaction among adolescents in other studies (12, 13, 17). Body image dissatisfaction was higher among overweight and underweight adolescent compared to those adolescent with normal body weight. This can be due to those underweight and overweight adolescents may think about their body weight repetitively which may cause body image dissatisfaction.

This study has some limitations. The major limitation of this cross-sectional study was that it did not show a temporal relationship between independent and dependent variables. In addition, because it used secondary data, some important factors such as self-esteem, school connectedness, family connectedness, sense of community, and autonomy were not assessed in this study. Therefore, we recommend further study on the effect of those factors that can be used to determine the level of body image satisfaction. With this limitation, this study provides evidence for this problem at the study site. In addition, it can be used as an input along with other similar studies in conducting systematic reviews and meta-analyses to produce a pooled estimate, especially for low-income countries, as there is little evidence regarding the problem in these countries, including Ethiopia.

## Conclusion

Almost One in five secondary school adolescents in Harar experienced body image dissatisfaction. Body image dissatisfaction was significantly associated with perception of being fat, anxiety, and cigarette smoking. In this study, a significant number of the adolescents experienced body image dissatisfaction. Hence, all concerned bodies have to take action to reduce the burden, and further research is also recommended especially by addressing those factor which were not addressed in this study.

## Data availability statement

The data set used and analyzed during the current study is available from the corresponding author upon reasonable request.

## Ethics statement

This study used data collected after obtaining ethical approval from the Institutional Health Research Ethics Review Committee of Haramaya University College of Health and Medical Sciences. The committee approved this study through the full consensus of all nine members of the IHRERC on June 21, 2022, with Ref. No. IHRERC/114/2022, in accordance with the Helsinki Declaration. Before data collection, informed, voluntary, written, and signed consent was obtained from each participant and their parents or guardians. For those aged below 18 years, verbal assent was obtained from adolescents before data collection. Only adolescents who provided written and signed consent participated in the study.

## Author contributions

AbT: Conceptualization, Formal analysis, Investigation, Methodology, Supervision, Writing – original draft, Writing – review & editing. LR: Formal analysis, Methodology, Writing – original draft, Writing – review & editing. MM: Formal analysis, Investigation, Methodology, Writing – original draft, Writing – review & editing. SS: Conceptualization, Formal analysis, Methodology, Writing – original draft, Writing – review & editing. OK: Methodology, Software, Supervision, Writing – original draft, Writing – review & editing. FS: Data curation, Investigation, Validation, Writing – original draft, Writing – review & editing. TD: Investigation, Methodology, Software, Writing – original draft, Writing – review & editing. AM: Investigation, Supervision, Validation, Writing – original draft, Writing – review & editing. AkT: Formal analysis, Investigation, Methodology, Writing – original draft, Writing – review & editing. KR: Investigation, Methodology, Supervision, Validation, Writing – original draft, Writing – review & editing.
